# Increased miR-142 Levels in Plasma and Atherosclerotic Plaques from Peripheral Artery Disease Patients with Post-Surgery Cardiovascular Events

**DOI:** 10.3390/ijms21249600

**Published:** 2020-12-16

**Authors:** Teodora Barbalata, Oriana E. Moraru, Camelia S. Stancu, Yvan Devaux, Maya Simionescu, Anca V. Sima, Loredan S. Niculescu

**Affiliations:** 1 Lipidomics Department, Institute of Cellular Biology and Pathology “Nicolae Simionescu” of the Romanian Academy, 8, B.P. Hasdeu Street, 050568 Bucharest, Romania; teodora.barbalata@icbp.ro (T.B.); camelia.stancu@icbp.ro (C.S.S.); maya.simionescu@icbp.ro (M.S.); anca.sima@icbp.ro (A.V.S.); 2 Emergency Clinical Hospital “Prof. Dr. Agrippa Ionescu”, 149 I.C. Brătianu Street, 077015 Baloteşti, Ilfov County, Romania; orianaelenamoraru@yahoo.com; 3 Cardiovascular Research Unit, Luxembourg Institute of Health, L-1445 Strassen, Luxembourg; yvan.devaux@lih.lu

**Keywords:** microRNA, pri-miRNA, peripheral artery disease, biomarker, cardiovascular event, femoral artery atherosclerotic plaque, bioinformatics

## Abstract

There is an intensive effort to identify biomarkers to predict cardiovascular disease evolution. We aimed to determine the potential of microRNAs to predict the appearance of cardiovascular events (CVEs) in patients with peripheral artery disease (PAD) following femoral artery bypass surgery. Forty-seven PAD patients were enrolled and divided into two groups, without CVEs (*n* = 35) and with CVEs (*n* = 12), during 1 year follow-up. Intra-surgery atherosclerotic plaques from femoral arteries were collected and the levels of miR-142, miR-223, miR-155, and miR-92a of the primary transcripts of these microRNAs (pri-miRNAs), and gene expression of *Drosha* and *Dicer* were determined. Results showed that, in the plaques, miR-142, miR-223, and miR-155 expression levels were significantly increased in PAD patients with CVEs compared to those without CVEs. Positive correlations between these miRNAs and their pri-miRNAs levels and the *Dicer/Drosha* expression were observed. In the plasma of PAD patients with CVEs compared to those without CVEs, miR-223 and miR-142 were significantly increased. The multiple linear regression analyses revealed significant associations among several plasma lipids, oxidative and inflammatory parameters, and plasma miRNAs levels. Receiver operator characteristic (ROC) analysis disclosed that plasma miR-142 levels could be an independent predictor for CVEs in PAD patients. Functional bioinformatics analyses supported the role of these miRNAs in the regulation of biological processes associated with atherosclerosis. Taken together, these data suggest that plasma levels of miR-142, miR-223, miR-155, and miR-92a can significantly predict CVEs among PAD patients with good accuracy, and that plasma levels of miR-142 can be an independent biomarker to predict post-surgery CVEs development in PAD patients.

## 1. Introduction

Despite medical and technological developments, cardiovascular diseases are still the main causes of death worldwide [1]. Atherosclerosis is a multifactorial, chronic, inflammatory disorder that affects medium and large arteries in various vascular beds [2]. It entails the accumulation of lipids in the arterial wall, a process that triggers a cascade of molecular and structural modifications that in the end leads to the obstruction of blood flow in the vessel, thrombus formation, and eventual rupture [3]. Peripheral artery disease (PAD) is an atherosclerotic disorder that affects arteries in the lower limbs and is one of the main causes of mortality and morbidity worldwide [4]. It is caused by the partial or total obstruction of the arteries (usually the femoral artery), which in turn leads to severe ischemic conditions in the limbs [5]. This process results in intermittent claudication, lesions, and even amputation of toes and limbs in the most extreme cases. Moreover, it has been reported that PAD patients are more likely to be affected by coronary artery disease (CAD) as well [6]. It is, therefore, vital to understand the molecular mechanisms and the predictive factors underlying the development and progression of PAD in order to find new therapeutic approaches.

MicroRNAs (miRNAs) are short noncoding RNAs approximately 22 nucleotides long involved in the control of gene expression. The biogenesis of miRNAs begins in the nucleus, where primary transcripts of miRNAs (pri-miRNAs) are cleaved by the Drosha enzyme, which turns them into precursor miRNAs (pre-miRs). Then, they are transported into the cytoplasm where they are cleaved a second time by the Dicer enzyme, thus being transformed into mature miRNAs, which can later degrade their target messenger RNAs (mRNAs) [7]. The miRNAs have been described to regulate various cellular processes in the pathophysiology of cancer [8], autoimmune diseases [9,10], cardiovascular diseases, and many others [11]. Moreover, miRNAs are found in various tissues and in the bloodstream, with their circulating levels being proposed as potential biomarkers to diagnose cardiovascular events, such as acute myocardial infarction [12]. There are few data regarding miRNA levels in human atherosclerotic plaques [13,14]. To our knowledge, the correlations between miRNA plaque expressions and their plasma levels, as well as with their corresponding pri-miRNA plaque expressions, have not been reported yet.

In this study, we analyzed the levels of a set of four miRNAs (hsa-miR-142-3p, hsa-miR-223-3p, hsa-miR-155-5p, and hsa-miR-92a-3p) in the plasma and femoral atherosclerotic plaques of PAD patients and correlated the data with the post-surgery outcome of the patients (subsequent cardiovascular events). We selected this particular set of miRNAs on the basis of our previous studies and on data from the literature. Increased levels of miR-142 have been shown to be associated with cardiovascular events (CVEs), especially with myocardial infarction [15], while miR-155, a macrophage-specific miRNA, is associated with inflammation in the context of atherosclerosis, as well as with the severity of CAD [16,17]. MiR-223 has been widely studied and shown to be involved in inflammation [18] and in the regulation of the lipid metabolism [19], and it has been shown to be upregulated in unstable atherosclerotic plaques from CAD patients [20]. MiR-92a is enriched in endothelial cells, where it mediates its communication with macrophages, thus contributing to atherosclerosis progression [21].

Considering the severity of PAD affliction, there is a need for novel precise biomarkers that could predict its evolution to improve life quality and personalized treatment of the patients. We aimed at identifying whether and which miRNAs have the potential to predict new CVEs, since they have many features that make them an attractive class of biomarkers. Consequently, we analyzed plaque and plasma expression of hsa-miR-142-3p, hsa-miR-223-3p, hsa-miR-155-5p, and hsa-miR-92a-3p, and we correlated them with the PAD patients’ evolution. We report here that hsa-miR-142-3p can be considered a predictive biomarker for future CVEs.

## 2. Results

### 2.1. Main Plasma Parameters of PAD Patients

The clinical variables, plasma lipids, and other parameters of the 47 patients in the study are presented in Table 1. The patients were monitored for 1 year for new CVEs. Coronary and femoral artery stent implantations, any other heart or vascular surgical procedures, and toe or leg amputations were recorded as new CVEs. The results showed that, compared to PAD patients without CVEs, PAD patients with CVEs displayed lower plasma levels of apolipoprotein A-I (APOA-I) (1.6-fold, *p* = 0.028), APOA-I/APOE ratio (1.7-fold, *p* = 0.025), and paraoxonase 1 (PON1) activity (2.2-fold, *p* = 0.046) and higher levels of ceruloplasmin (CP) (1.5-fold, *p* = 0.002) and C-reactive protein (CRP) (2.4-fold, *p* = 0.022).

### 2.2. MiRNA, pri-miRNA, and mRNA Expressions in the Atherosclerotic Plaques Collected from the Femoral Arteries of PAD Patients

**Figure 1:**
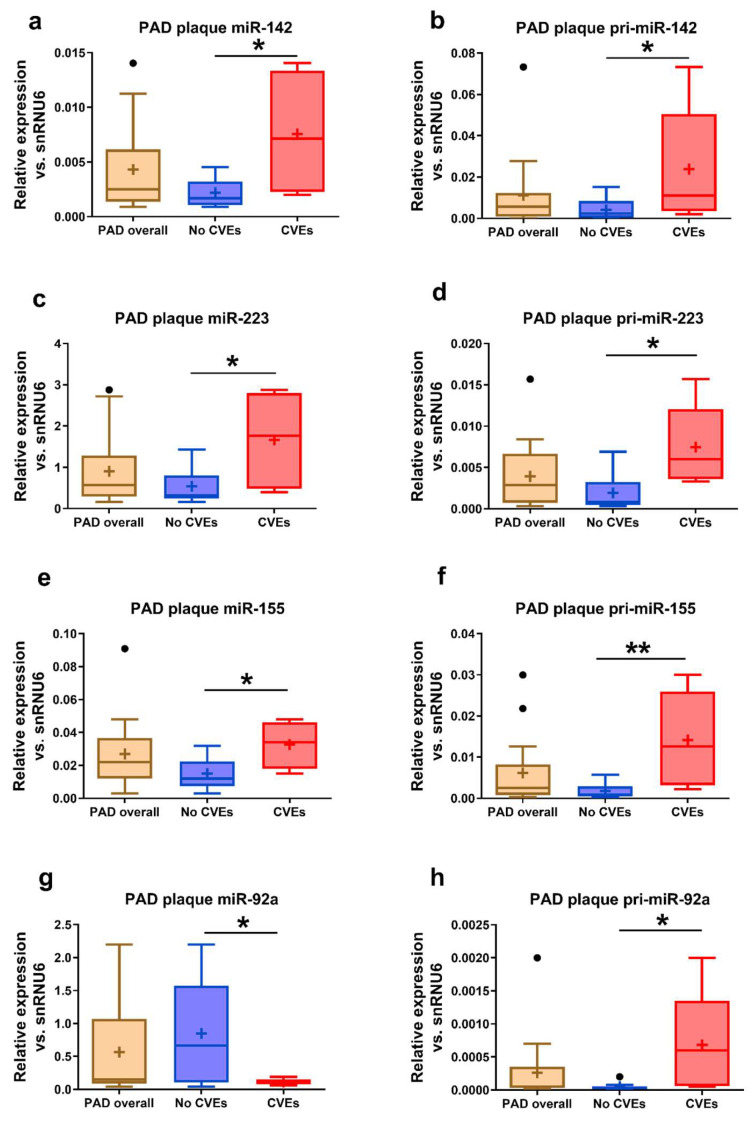
Levels of microRNAs (miRNAs) and their primary transcripts (pri-miRNAs) in the atherosclerotic plaques of peripheral artery disease (PAD) patients. Levels of miR-142 (**a**), miR-223 (**c**), miR-155 (**e**), miR-92a (**g**), pri-miR-142 (**b**), pri-miR-223 (**d**), pri-miR-155 (**f**), and pri-miR-92a (**h**) in the atherosclerotic plaques of PAD patients with cardiovascular events (CVEs) compared to those with no CVEs. Data are illustrated as boxplots with Tukey whiskers, median line, and mean cross (+). * *p* < 0.05, ** *p* < 0.01 vs. no CVEs.

Higher levels of miR-142 (3.4-fold, *p* = 0.029) (Figure 1a), miR-223 (3-fold, *p* = 0.021) (Figure 1c), and miR-155 (2.2-fold, *p* = 0.028) (Figure 1e) and lower levels of miR-92a (7.6-fold, *p* = 0.015) (Figure 1g) were determined in the atheromas from femoral arteries collected from PAD patients with CVEs compared to those without CVEs. To estimate the contributions of plaque miRNA production to plaque miRNA contents, we measured specific pri-miRNA expressions in the plaques. In good agreement with these data, higher levels of pri-miR-142 (5.8-fold, *p* = 0.021) (Figure 1b), pri-miR-223 (4-fold, *p* = 0.013) (Figure 1d), pri-miR-155 (7.8-fold, *p* = 0.008) (Figure 1f), and pri-miR-92a (13-fold, *p* = 0.020) (Figure 1h), were observed in the plaques of PAD patients with CVEs vs. those without CVEs.

**Figure 2:**
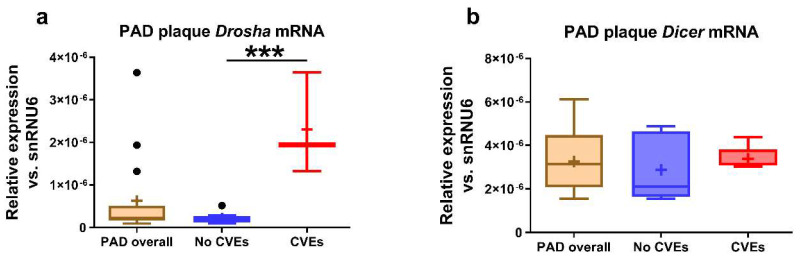
Gene expression of Dicer and Drosha in the plaques of peripheral artery disease (PAD) patients. The messenger RNA (mRNA) levels of Drosha (**a**) and Dicer (**b**) in the atherosclerotic plaques of PAD patients with cardiovascular events (CVEs) compared to those without CVEs. Data are illustrated as boxplots with Tukey whiskers, median line, and mean cross (+). *** *p* < 0.001 vs. no CVEs.

Higher expression of *Drosha* mRNA (10.8-fold, *p* = 0.0002) was detected in the femoral artery atheromas of PAD patients with CVEs compared to those without CVEs (Figure 2a), while no significant differences were observed for plaque *Dicer* mRNA expression (Figure 2b).

**Figure 3:**
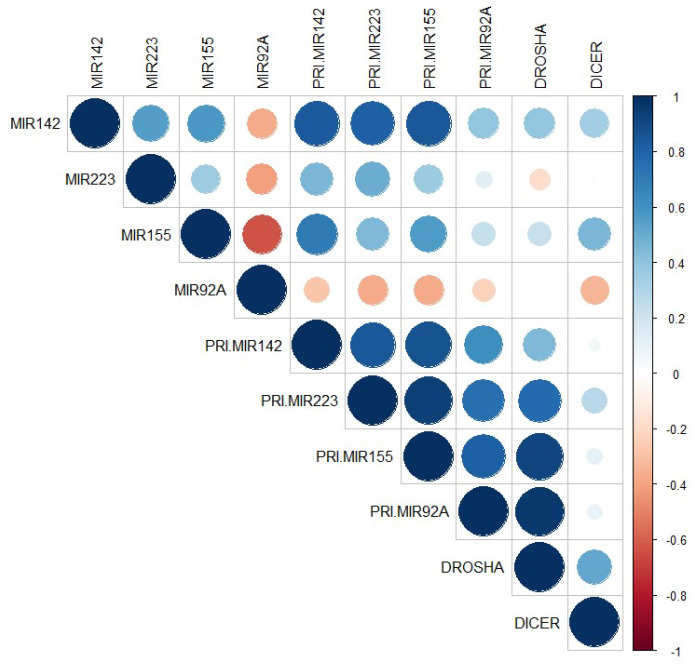
Bivariate correlations among miRNAs, pri-miRNAs, and mRNAs in the plaques of peripheral artery disease (PAD) patients using Spearman’s function.

Bivariate correlations among miRNAs, pri-miRNAs, and mRNAs in the plaques of PAD patients were estimated by Spearman’s function and are illustrated in Figure 3 (given in detail in Table S1, Supplementary Materials). We found a strong positive correlation between miR-142 and pri-miR-142 expressions in femoral artery atheromas from PAD patients (*R* = 0.837, *p* = 3.53 × 10^−4^) and a good positive correlation between plaque miR-155 and pri-miR-155 (*R* = 0.566, *p* = 0.035) expressions. Plaque miR-142 expression strongly correlated with pri-miR-223 (*R* = 0.814, *p* = 7.02 × 10^−4^) and with pri-miR-155 (*R* = 0.843, *p* = 2.96 × 10^−4^) expression. A strong correlation between plaque miR-155 and pri-miR-142 expression was also observed (*R* = 0.705, *p* = 0.007). Very strong correlations were determined between mRNA Drosha in the plaques and pri-miRNAs: pri-miR-223 (*R* = 0.778, *p* = 3.9 × 10^−4^), pri-miR-92a (*R* = 0.965, *p* = 1.47 × 10^−9^), and pri-miR-155 (*R* = 0.912, *p* = 8.61 × 10^−7^).

### 2.3. miRNA Levels in the Plasma of PAD Patients

**Figure 4:**
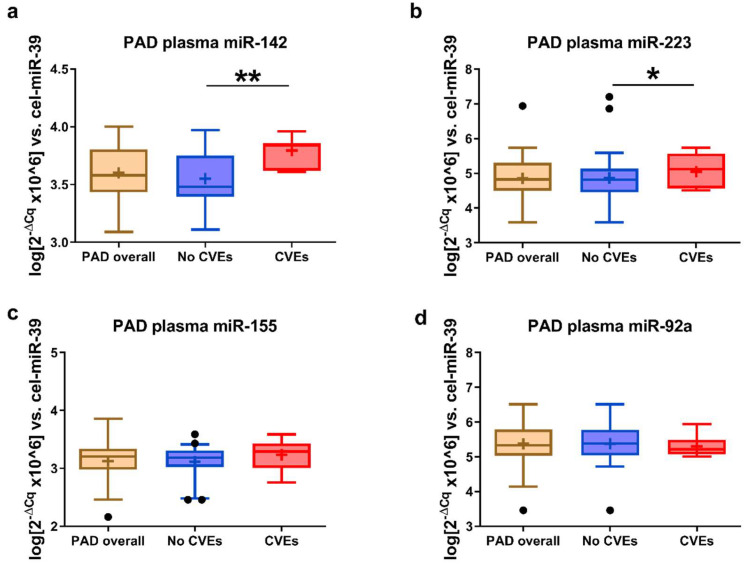
The miRNA levels in the plasma samples of peripheral artery disease (PAD) patients. Note the levels of miR-142 (**a**), miR-223 (**b**), miR-155 (**c**), and miR-92a (**d**) in the plasma of patients with new cardiovascular events (CVEs) vs. those without CVEs. Data are illustrated as boxplots with Tukey whiskers, median line, and mean cross (+). * *p* < 0.05, ** *p* < 0.01 vs. no CVEs.

In the plasma of PAD patients with CVEs compared to those without CVEs, significantly higher levels of miR-142 (1.6-fold, *p* = 0.013) (Figure 4a) and miR-223 (2-fold, *p* = 0.05) (Figure 4b) were determined, while no significant differences were found for miR-155 and miR-92a (Figure 4c,d).

**Figure 5:**
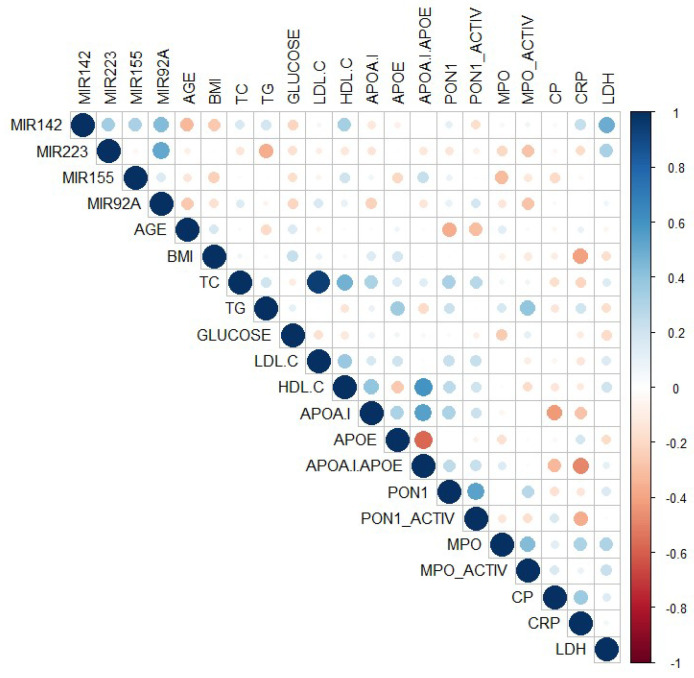
Bivariate correlations between plasma miRNA levels and main parameters using Spearman’s function.

Bivariate correlations between plasma miRNAs levels and the main parameters were estimated by Spearman’s nonparametric analysis and are illustrated in Figure 5 (given in detail in Table S2, Supplementary Materials). Plasma miR-223 levels negatively correlated with the triglyceride levels (*R* = −0.369, *p* = 0.015), while plasma miR-142 levels negatively correlated with the age of the patients (*R* = −0.328, *p* = 0.044) and positively correlated with lactate dehydrogenase (LDH) levels (*R* = 0.493, *p* = 0.008).

PAD patients’ main parameters were applied in a multiple regression analysis to statistically estimate the variance of plasma miRNA levels. Multiple linear regression (MLR) models that included clinical variables (age, gender, body mass index (BMI), obesity, hypertension, dyslipidemia, and hyperglycemia), blood lipids (total cholesterol (TC) and triglyceride (TG) levels, high-density lipoprotein cholesterol (HDL-C)/low-density lipoprotein cholesterol (LDL-C), and APOA-I/APOE ratios), and oxidative and inflammation parameters (PON1 levels, PON1 activity, myeloperoxidase (MPO) protein, MPO activity, CP, and CRP levels) gave statistically significant predictions (estimated from adjusted *R*-squared values) for the variance of plasma miRNA levels (Table 2). Accordingly, MLR model 1 (clinical data) could significantly predict 84.5% of the PAD patients’ plasma levels variance of miR-142 (*p* < 0.001), 72.3% for miR-155 (*p* < 0.001), and 51.7% for miR-92a (*p* < 0.001). In this MLR model 1, BMI was a strong predictor for plasma miR-142 level variance (β = 0.908, *p* = 0.025), while dyslipidemia could significantly predict miR-142 (β = −0.329, *p* = 0.045) and miR-92a (β = −0.949, *p* = 0.002) variance.

The multiple linear regression (MLR) analysis using blood lipids (model 2) significantly predicted the PAD patients’ plasma level variance of miR-142 (85.0%, *p* < 0.001), miR-155 (75.1%, *p* < 0.001), miR-92a (39.6%, *p* < 0.001), and, to a lesser extent, miR-223 (20.6%, *p* = 0.018), suggesting a strong association of all analyzed miRNAs with the lipid metabolism (Table 2). Among the individual predictors in the model 2, HDL-C/LDL-C ratio significantly predicted plasma variance for miR-223 (β = 1.007, *p* = 0.004), miR-155 (β = 0.588, *p* = 0.003), and miR-142 (β = 0.376, *p* = 0.012) levels, while TC could significantly predict miR-142 variance (β = 0.584, *p* = 0.002).

Oxidative and inflammatory parameters (MLR model 3) of PAD patients were significantly associated with the variance of plasma levels of miR-142 (84.7%, *p* < 0.001), miR-155 (67.1%, *p* < 0.001), and miR-92a (40.1%, *p* < 0.001) (Table 2). In this MLR model 3, plasma level variance of miR-142 was significantly associated with levels of CP (β = 0.645, *p* < 0.001), PON1 (β = 0.300, *p* = 0.044), and MPO (β = 0.572, *p* = 0.015).

Other parameters of PAD patients, such as LDH, alanine transaminase (ALT), and aspartate transaminase (AST) levels (MLR model 4), could significantly predict the plasma level variance of miR-142 (85.3%, *p* < 0.001), miR-155 (71.2%, *p* < 0.001), miR-92a (34.4%, *p* < 0.001), and, to a lesser extent, miR-223 (14.7%, *p* = 0.039) (Table 2). In this MLR model 4, LDH could predict the plasma variance of miR-142 (β = 0.556, *p* < 0.001) and miR-155 (β = 0.453, *p* = 0.009), while ALT levels significantly supported the plasma variance of miR-142 (β = 0.324, *p* = 0.018) and miR-223 (β = 0.766, *p* = 0.020).

### 2.4. Associations of Plasma miRNAs Levels with CVEs in Post-Surgery PAD Patients

Binary logistic regression (BLR) modeling was used to estimate the appearance of CVEs in PAD patients using their plasma miRNAs levels as potential predictors. Using the plasma levels of all four analyzed miRNAs, the unadjusted BLR model could significantly predict the new CVEs during the 1 year post-surgery follow-up of PAD patients (χ^2^ = 24.75, *p* < 0.001) with an accuracy of 86.7% (Table 3). The significant contribution to this unadjusted BLR model was given by plasma levels of miR-142 (Wald χ^2^ = 4.58, *p* = 0.032) and miR-92a (Wald χ^2^ = 5.29, *p* = 0.021). When adjusted for age and gender (with male patients, as risk group), the BLR model remained a significant predictor for new CVEs in PAD patients (χ^2^ = 25.47, *p* < 0.001) and the accuracy of the model stayed at 86.7% (Table 3). As expected, the significant contributors to the BLR model remained the combination of miR-142 (Wald χ^2^ = 3.19, *p* = 0.034) and miR-92a (Wald χ^2^ = 3.97, *p* = 0.046) plasma levels. An incomplete BLR model with at least two or three plasma miRNAs levels proved to be without statistical significance, both in adjusted and in unadjusted models (data not shown).

We then estimated the prediction potential of each of the four plasma miRNAs for new CVEs in PAD patients by using receiver operator characteristic (ROC) curve analysis. According to this analysis, only the plasma levels of miR-142 proved to be a strong independent predictor of new CVEs in PAD patients (area under the curve (AUC) = 0.861, *p* = 0.007), while the other miRNAs failed to reach the statistical significance (Table 4). However, the combination of all four analyzed miRNAs could significantly predict the CVEs in PAD patients, in multivariate ROC curve analysis, both unadjusted (AUC = 0.910, *p* = 0.002) and adjusted for age and gender (AUC = 0.924, *p* = 0.002).

### 2.5. Functional Analyses—Gene Ontology (GO) Biological Processes Targeted by the Set of Four miRNAs

**Figure 6:**
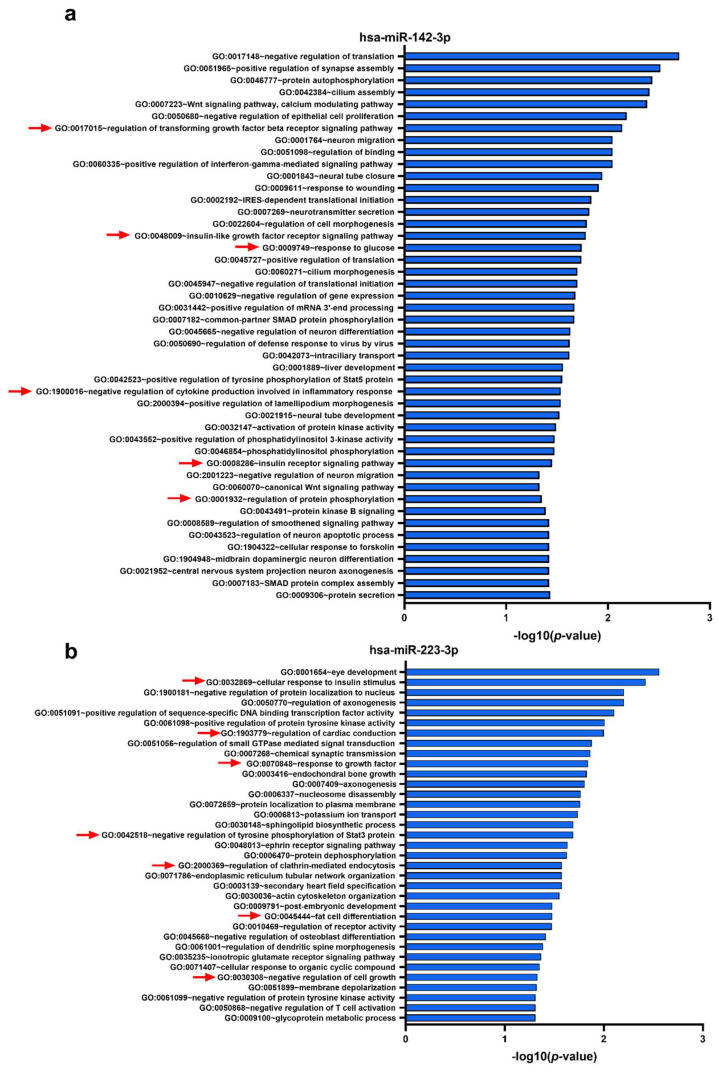
Database for Annotation, Visualization, and Integrated Discovery (DAVID) functional analysis for Gene Ontology (GO) biological processes associated with target genes identified by miRWalk algorithm to be potentially regulated by the selected microRNAs, i.e., miR-142-3p (**a**) and miR-223-3p (**b**). Selected GO biological process terms are indicated by red arrows.

The miRWalk algorithm predicted 647, 2169, 759, and 473 individual human genes potentially targeted by hsa-miR-142-3p, hsa-miR-223-3p, hsa-miR-155-5p, and hsa-miR-92a-3p, respectively. We then performed biological process enrichment analysis for the identified target genes of each miRNA using the Database for Annotation, Visualization, and Integrated Discovery (DAVID) platform, applying a double filtering for unadjusted *p-*values less than 0.05 and a fold enrichment over 2. The gene targets of hsa-miR-142-3p, which was the only miRNA proven to be an independent predictor for new CVEs in PAD patients and upregulated in the femoral artery plaques and plasma from PAD patients with CVEs, are associated with 46 biological processes including the Wnt signaling pathway, calcium modulating pathway, regulation of transforming growth factor β receptor signaling pathway, positive regulation of interferon γ-mediated signaling pathway, insulin-like growth factor receptor signaling pathway, response to glucose, negative regulation of cytokine production involved in inflammatory response, insulin receptor signaling pathway, and regulation of protein phosphorylation (Figure 6a). A total of 35 biological processes were found to be regulated by the targets of hsa-miR-223-3p, which was upregulated in the femoral artery atherosclerotic plaques and plasma of PAD patients with CVEs, with some of them being associated with cellular response to insulin, regulation of cardiac conduction, response to growth factors, negative regulation of tyrosine phosphorylation of Stat3 protein, regulation of clathrin-mediated endocytosis, fat cell differentiation, and negative regulation of cell growth (Figure 6b).

**Figure 7:**
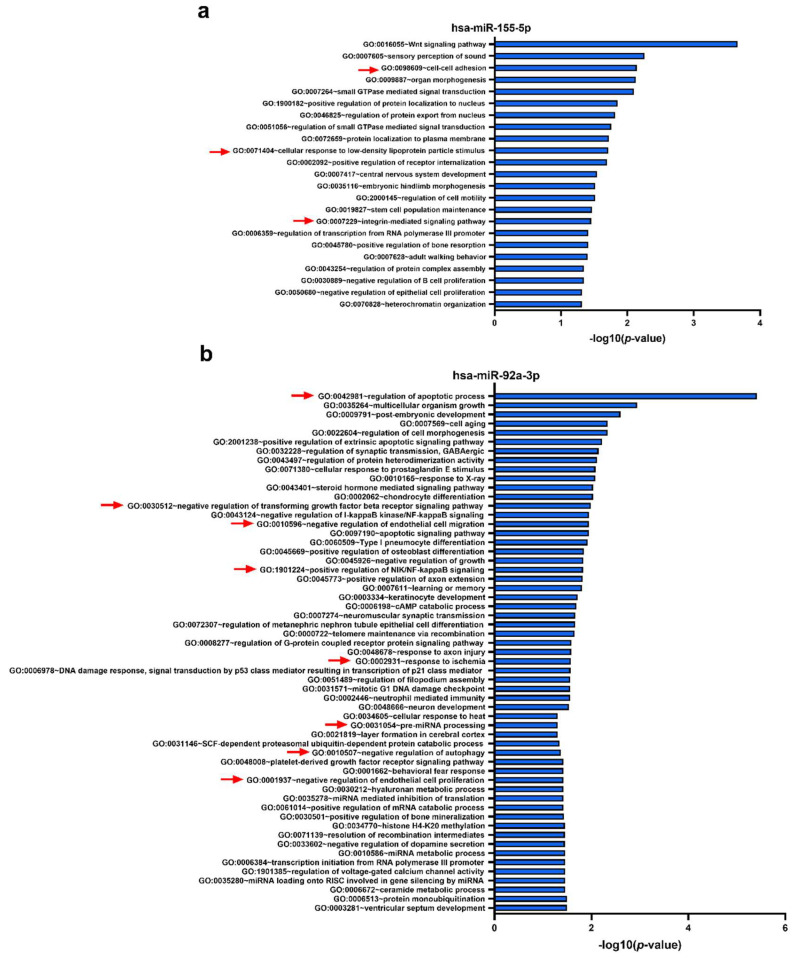
DAVID functional analysis for Gene Ontology (GO) biological processes associated with target genes identified by miRWalk algorithm to be potentially regulated by the selected microRNAs, i.e., miR-155-5p (**a**) and miR-92a-3p (**b**). Selected GO biological process terms are indicated by red arrows.

The potential targets of hsa-miR-155-5p, whose levels were increased in plaques from PAD patients with CVEs, may potentially regulate 23 biological processes, some of them being associated with cell–cell adhesion, cellular response to low-density lipoprotein particles, and integrin-mediated signaling pathway (Figure 7a). Lastly, hsa-miR-92a-3p, which was downregulated in femoral artery plaques and upregulated in plasma from PAD patients with CVEs, was found enriched in 57 biological processes including the regulation of apoptosis, negative regulation of transforming growth factor β receptor signaling pathway, negative regulation of endothelial cell migration, positive regulation of nuclear factor-κB (NF-κB)/NF-κB-inducing kinase) signaling, response to ischemia, pre-miRNA processing, negative regulation of autophagy, and negative regulation of endothelial cell proliferation (Figure 7b).

## 3. Discussion

In this study, we assessed the potential of miRNAs to predict post-surgery CVEs in PAD patients by measuring miRNAs levels in the plasma and femoral atherosclerotic plaques and correlated the data with newly occurred CVEs during 1 year. The novel data showed that, in comparison with PAD patients without CVEs, PAD patients with CVEs displayed: (i) significant increases in miR-142, miR-223, and miR-155 expressions in atherosclerotic plaques, which correlated positively with the pri-miRNAs levels, (ii) significant increases in plasma miR-142 and miR-223 levels, (iii) a significantly decreased plaque miR-92a expression, while pri-miR-92a expression was increased, and (iv) significant associations between several blood lipids, oxidative and inflammatory parameters, and the plasma miRNAs levels, as evidenced in multiple regression analysis models. Furthermore, plasma levels of the four miRNAs analyzed could significantly predict CVEs with good accuracy by binary logistic regression, with miR-142 and miR-92a being the significant predictors in this model, and plasma miR-142 was found to be an independent predictor for CVEs in PAD patients. To our knowledge, the present study is the first report on the prediction of disease evolution using a comparative analysis of miRNA levels in plasma and atherosclerotic plaque in PAD patients. Moreover, we show for the first time correlations between atherosclerotic plaque miRNA contents and specific pri-miRNA plaque expressions, enabling the cross-plaque gradient analysis with the circulating cell-free miRNA levels.

An early and accurate prognosis for the disease evolution in PAD patients can optimize clinical decisions to better adjust interventions for individual patients. To put the promise of personalized medicine in practice, miR-142 can contribute to risk stratification in PAD patients by predicting CVEs, making translational research feasible. Our novel data raise the possibility for the use of miRNA-targeted therapy (i.e., in vivo inhibition of miR-142) to assist the statin-based atherosclerosis treatment by modulating the miRNA-related mechanisms.

PAD is a vascular problem of diffuse atherosclerosis and might contribute to cardiovascular morbidity and mortality. Many studies have tried to gain insight into molecular mechanisms involved in PAD. Currently, highly stable circulating cell-free small noncoding RNAs, such as miRNAs, seem to have disease-specific expressions. Thus, miRNAs are considered new measurable prognostic, noninvasive biomarkers and a start point for individualized treatment. To estimate the potential of plasma miRNAs to predict CVEs appearance in PAD patients, we selected four miRNAs, miR-223-3p, miR-92a-3p, miR-142-3p, and miR-155-5p, and analyzed their expression levels in plasma and femoral atherosclerotic plaques. This selection was based on our previously published data demonstrating that plasma miR-223 and miR-92a levels are increased in hyperlipidemic and/or hyperglycemic CAD patients [22]. Moreover, our preliminary unpublished screening data in atherosclerotic plaques show that miR-142 and miR-155 have high levels of expression, which correlate with recent reports associating these two miRNAs with CAD severity and major cardiovascular events [15,16].

The most valuable and novel result of our study is the potential of plasma miR-142-3p to independently predict the post-surgery appearance of CVEs in PAD patients. Recently, high plasma miR-142 levels were associated with a significantly higher risk of major adverse CVEs in dual antiplatelet-treated patients undergoing percutaneous coronary intervention, independently of diabetes mellitus, heart failure, and calcium channel blocker application [15]. Xu et al. demonstrated that miR-142 is significantly increased in atherosclerotic plaques of *apoe*^−/−^ mice following a high-fat diet and the upregulation of miR-142 expression determined apoptosis in human macrophages by targeting the transforming growth factor (TGF)-β2 [23]. These data are in good agreement with our results, showing an increased miR-142 expression in the femoral atherosclerotic plaques from PAD patients with CVEs compared to those without CVEs. This increased plaque expression of miR-142 positively correlates with plaque pri-miR-142 and plasma miR-142 levels, suggesting a local vascular production of miR-142 in the atheroma and its possible secretion in the blood. Another study shows that hypoxia-associated overexpression of miR-142 produces extensive cell injury and apoptosis, while miR-142 suppression significantly promotes cell viability and attenuates apoptosis [24]. Recently published data show that miR-142 downregulation inhibits human aortic smooth muscle cell (SMC) proliferation and migration, possibly by targeting myocardin-like protein 2 [25]. Furthermore, the overexpression of miR-142 may induce the proliferation of human vascular SMC, by targeting B-cell translocation gene 3 which regulates vascular cell proliferation [26]. In addition, it has been shown that miR-142 is a key regulator of cardiomyocyte fibrosis and apoptosis induced by hypoxia/reoxygenation, thus serving as a candidate for myocardial injury therapeutic strategy [9]. Qin et al. found that miR-142 is an essential mediator for endothelial cells (ECs) apoptosis both in vitro and in vivo, being upregulated in ox-LDL-induced apoptotic human aortic ECs by inhibiting the protein kinase B (Akt)/endothelial nitric oxide synthase signaling pathway [27]. Additionally, they showed that systemic treatment with the miR-142-3p antagomir attenuates EC apoptosis and delays the progression of atherosclerosis in the aorta of *apoe*^−/−^ mice. Taken together, these data testify for an important role of miR-142 in the regulation of key processes in the main vascular wall cells associated with the development of atherosclerotic plaques. Our data show that the plasma levels of miR-142 in PAD patients correlate with some clinical features (age, BMI), blood lipids, and oxidative and inflammatory parameters, suggesting a possible connection with these metabolic processes.

Another miRNA investigated in our study is miR-223-3p, which was recently proven to be a circulating biomarker for plaque instability both in serum and in tissue (atherectomy and thrombectomy material), being related to the acute phase of CAD [20]. Some studies have shown that miR-223 could be released from the vascular ECs and that it might play a role in EC dysfunction, atherosclerosis initiation, progression, and possibly plaque rupture [28,29]. Therefore, similar to miR-142-3p, miR-223-3p might originate within the plaque and directly reflect damage of EC in the plaque on the edge of rupture. This hypothesis is supported by the observation that the plaque miR-223 expression is positively correlated with plaque pri-miR-223 expression and with plasma miR-223 levels. Wang et al. showed that miR-223 may reduce lipid accumulation in macrophages by activating the phosphatidylinositol 3-kinase/protein kinase B pathway, suggesting a direct connection between miR-223 and the cholesterol metabolism [30]. Our data show that plasma miR-223 levels correlate with blood lipids, in good agreement with Vickers’s report about its regulatory potential in the cholesterol metabolism [31]. Another study shows that miR-223 promotes human umbilical vascular EC apoptosis by targeting the insulin-like growth factor 1 receptor [29]. In contrast, Li et al. demonstrated that miR-223 can negatively regulate the coagulation cascade by inhibiting tissue factor expression in human vascular aortic EC, suggesting its protective role against thrombogenesis during the process of atherosclerotic plaque rupture [32]. As far as we know, our data prove for the first time that blood circulating cell-free miR-223 may originate from atherosclerotic plaques, but its plasma levels do not associate with post-surgery CVEs in PAD patients. These results are partly in contrast with other data that show a strong correlation of serum miR-223 with the acute phase of CAD [20]. We show that plasma miR-223 levels are increased in PAD patients with CVEs, but BLR and ROC analysis did not indicate it as an independent biomarker.

The third miRNA in the set analyzed in our study is miR-155-5p; recently published data showed that serum miR-155 level is associated with classical risk factors of atherosclerotic lesions (positive correlation with Gensini score) and may serve as a biomarker for evaluating the severity of CAD [16]. Our data show that the atherosclerotic plaque expression of miR-155 is higher in PAD patients with CVEs compared to those without CVEs and positively correlates with plaque pri-miR-155 expression. However, in contrast with Qiu and Ma group’s data on CAD patients [16], we detected no variation in PAD patients’ plasma when comparing the subgroups associated with the post-surgery CVEs. In good agreement with our data on PAD atherosclerotic plaques, other studies showed that miR-155 is upregulated in human cardiac diseases [33] and is significantly increased in plasma and plaques from CAD patients [34]. Our results indicate that plasma miR-155 levels correlate with clinical data, blood lipids, and oxidative and inflammatory parameters of PAD patients. In contrast, Zhu et al. showed that miR-155 levels in plasma are lower in patients with unstable angina pectoris and acute myocardial infarction than in patients with chest pain syndrome. The same study reported that miR-155 levels are inversely associated with proatherogenic metabolic risk factors (age, hypertension, total cholesterol, and HDL-C, LDL-C, and CRP levels) and the severity of atherosclerotic lesions [35].

Previous studies indicated that miR-92a-3p may induce ECs dysfunction and the development of coronary atherosclerotic lesions [36]. Inhibition of miR-92a may reduce and stabilize atherosclerotic plaques [37]. The group of Stojkovic et al. showed that serum miR-92a-3p is an independent predictor of lesion restenosis and atherothrombotic events (primary endpoint) after adjustment for age, sex, and clinical risk factors, but fails to predict the target vessel revascularization (secondary endpoint) in PAD patients after infra-inguinal angioplasty with stent implantation [38]. These data are partly confirmed by our data, showing that post-surgery CVEs in PAD patients are associated with decreased miR-92a expression and increased pri-miR-92a expression in femoral atherosclerotic plaques, while its levels do not vary in plasma. However, we show that plasma miR-92a level can significantly predict CVEs in PAD patients, but only together with miR-142, miR-223, and miR-155 levels in a binary logistic model after adjusting for age and sex. In addition, ROC analysis does not indicate miR-92a as an independent predictor for new CVEs in PAD patients. In vitro functional data show that miR-92a induces ECs inflammation and may promote vascular SMC proliferation and restenosis [39]. Furthermore, miR-92a inhibition enhances ECs migration, proliferation, and angiogenesis and prevents atherosclerosis and restenosis in vivo, in experiments on rats with carotid artery balloon injury, mice with limb ischemia and myocardial infarction, and mice with wire-induced injury of the femoral artery [37,40,41]. We found that plasma miR-92a levels correlate with dyslipidemia and oxidative and inflammatory parameters of PAD patients.

Concerning post-surgery events in atherosclerosis evolution, there are reports that some specific molecules can be used as potential biomarkers for both development and post-surgery course of atherosclerosis. For example, Zinellu et al. suggested that plasma asymmetric dimethylarginine levels were associated with carotid narrowing after interventional endarterectomy [42]. However, these newly emerging molecules, along with miRNAs, need further investigations for their prognosis potential for the disease evolution in PAD patients, together with the established biomarkers and current imagistic investigation already used in clinics.

A limitation of our study is the relatively small number of PAD patients analyzed. Nevertheless, although the number of PAD patients recruited was rather small, the employed statistical analyses and models, as well as the distribution of the studied miRNAs set in the femoral atherosclerotic plaques and plasma, revealed statistically significant data. We also explored the contribution of some important risk factors (age, gender, BMI, obesity, hypertension, dyslipidemia, and hyperglycemia) considering them cofactors in the statistical models. Another drawback of our miRNA study is that, despite the various techniques used, there are no standardized methods to quantify the absolute levels of blood cell-free circulating (plasma compartment) miRNAs, making the comparison among results from different laboratories difficult.

In conclusion, we demonstrated that miR-142, miR-223, miR-155, and miR-92a are produced in the femoral atherosclerotic plaques as ascertained by their specific pri-miRNA expression and positive correlations with key components of the miRNAs processing complex (Dicer, Drosha). We also showed that miR-142, miR-223, miR-155, and miR-92a plaque expressions are associated with post-surgery CVEs in PAD patients and correlate with the proatherogenic metabolic, oxidative, and inflammatory risk factors. Lastly, according to the data obtained from statistical regression modeling, we propose plasma miR-142 as an independent biomarker to predict CVEs in PAD patients.

## 4. Materials and Methods 

### 4.1. Study Design and Subjects

The study included 47 patients (four women and 43 men) diagnosed with PAD aged between 32 and 83 years old, subjected to a femoral artery bypass procedure. The patients were monitored for 1 year for new CVEs and were divided according to this criterion into two groups: PAD patients with new CVEs (*n* = 12) and PAD patients without new CVEs (*n* = 35). We considered new CVEs any of the following: coronary artery stent implantation, any other heart or vascular surgical procedures, and toe or leg amputations. All PAD patients were enrolled at the Emergency Clinical Hospital “Prof. Dr. Agrippa Ionescu”, Balotesti, Ilfov County, Romania. Upon admission to hospital, plasma samples were collected from all PAD patients and, later on, during the bypass procedure, a sample of the removed atherosclerotic plaque was collected for the study. Tissue samples were stored in DNA/RNA Shield (Zymo Research, Irvine, CA, USA) immediately after collection until processing. None of the patients received heparin or fractionated heparin at the time of plasma sampling.

This study was carried out in accordance with the principles from the Declaration of Helsinki (The Code of Ethics of the World Medical Association, last updated at the 64th WMA General Assembly, Fortaleza, Brazil, October 2013) for experiments involving humans. All participants gave their written informed consent by signing the appropriate paperwork and respecting their anonymity and privacy rights; they were allocated a number in the study database. The Ethics Committees of the Institute of Cellular Biology and Pathology “Nicolae Simionescu” (authorization number 8, on 27 June 2016) and of the Emergency Clinical Hospital “Prof. Dr. Agrippa Ionescu” (authorization number 1367695, on 16 June 2016) approved the study.

### 4.2. Determination of Plasma Parameters

Plasma levels of total cholesterol, HDL-C, LDL-C, triglycerides, glucose, LDH, and hepatic transaminases (ALT and AST) were determined by the hospital’s laboratory. The plasma parameters presented in Table 1 were measured using the following commercially available kits: APOA-I and APOE (Mabtech, Nacka Strand, Sweden), PON1 and MPO (R&D Systems, Minneapolis, MN, USA), MPO activity (Cayman Chemical, Ann Arbor, MI, USA), CP (Abnova, Taipei, Taiwan), and CRP (Wako Chemicals GmbH, Neuss, Germany). All assays were performed according to the manufacturer’s instructions. PON1 activity was measured using an adapted method in the serum of the patients [43].

### 4.3. Analysis of microRNA in the Plasma and Atherosclerotic Plaques

The miRNeasy Serum/Plasma kit (Qiagen, Hilden, Germany) was used to isolate miRNAs from plasma, following the manufacturer’s instructions. During miRNA purification, we added 25 fmol of synthetic cel-miR-39 (Life Technologies, Carlsbad, CA, USA) to each sample as a spike-in to correct the sample-to-sample variation, as previously described [22,44]. MiRNAs were eluted in 22 μL of RNase-free water and stored at −80 °C until further analysis. Total RNA was extracted from the atherosclerotic plaques using TRIzol reagent (Thermo Scientific, Waltham, MA, USA), according to the manufacturer’s protocol, resuspended in 20 μL of RNase-free water, and stored at −80 °C. 

The plasma and plaque expressions of hsa-miR-142-5p (ID 000464), hsa-miR-223-3p (ID 002295), hsa-miR-155-5p (ID 002623), and hsa-miR-92a-5p (ID 000431) were measured using TaqMan technology (Thermo Scientific, Waltham, MA, USA) as we previously reported [22,44].The expression level of each individual miRNA was determined relative to that of exogenously added cel-miR-39 (ID 000200, for plasma) or snRNU6 (ID 001973, for plaques) and calculated by using the 2^−Δ*C*q^ method [44].

The plaque expressions of pri-miR-142 (ID Hs03303162_pri), pri-miR-223 (ID Hs03303017_pri), pri-miR-155 (ID Hs03303349_pri), and pri-miR-92a (ID Hs03295977_pri) were measured by TaqMan technology, using random primers (Applied Biosystems, Foster City, CA, USA) for the reverse-transcription reaction (on Veriti PCR machine, Applied Biosystems, Foster City, CA, USA) and TaqMan technology normalizing against snRNU6 (using ViiA7 real-time PCR system, Applied Biosystems, Foster City, CA, USA). The expression level of each individual pri-miRNA was determined relative to that of snRNU6 and calculated by using the 2^−Δ*C*q^ method [44]. Plaque mRNA levels for *Drosha* and *Dicer* were measured and normalized against *18S*, using custom primers (Table S3, Supplementary Materials) and the SyBr Green method (SyBr Select master mix, Thermo Scientific, Waltham, MA, USA).

### 4.4. Functional Analysis of miRNAs

The potential miRNA target genes were predicted using miRWalk database version 3 (Medical Faculty Mannheim, University of Heidelberg, Heidelberg, Germany, http://mirwalk.umm.uni-heidelberg.de) [45]. For Gene Ontology biological process enrichment analysis, we used the DAVID (The Database for Annotation, Visualization and Integrated Discovery) knowledgebase platform version 6.8 (Frederick National Laboratory for Cancer Research, Frederick, MD, USA, https://david.ncifcrf.gov) [46]. We selected only the Gene Ontology (GO) biological process terms with an unadjusted *p-*value < 0.05 and a fold enrichment > 2.

### 4.5. Statistical Analysis

Statistical analysis was done using the statistical software SPSS for Windows version 21.0 (IBM SPSS, IBM Ireland, Dublin, Ireland). Graphical representations were done using GraphPad Prism version 9.0 (GraphPad Software Inc., San Diego, CA, USA), Statistical Software Package R 4.0.3 (particularly xlsx and corrplot packages), and R-studio for Windows version 1.3.1093 (RStudio PBC., Boston, MA, USA). The continuous distributed quantitative variables (biochemical, miRNA, and pri-miRNA data) were expressed as means ± standard error of the mean (SEM) and analyzed by Mann–Whitney U-test for the comparison between “no CVEs” and “CVEs”. Crosstab distribution with chi-squared (χ^2^) analysis was performed by SPSS to evaluate the differences between categorical variables (gender, age distribution, presence of obesity, hyperglycemia, hypertension, or dyslipidemia). For some analysis, the values of plasma miRNAs levels were log-transformed. Spearman’s nonparametric bivariate correlation analysis was performed using SPSS for plaque and plasma miRNAs levels with clinical and biochemical parameters. A similar correlation analysis was done for plaque pri-miRNAs, miRNAs, and Dicer/Drosha expression. Multiple linear regression models of the plasma miRNA levels (as a dependent variable) with blood lipids (model 1), oxidative and inflammation parameters (model 2), pathological parameters (model 3), and clinical data (model 4), as independent variables, were estimated, and the significant adjusted *R*-squared estimates were used as a prediction score for the plasma miRNA variance, while β coefficients predicted each parameter’s contribution to the model. To analyze the potential of plasma miRNAs to predict follow-up CVEs, we employed a binary logistic regression (BLR) model with the enter iteration method, considering the “no CVEs” group as the reference category and the “CVEs” group as the risk (vulnerable) category, adjusting for age and gender (with females as reference group). Receiver operator characteristic (ROC) curve analysis (two-value) was used to estimate the prediction ability of a follow-up CVEs for plasma miRNAs levels; multivariate ROC curve analysis was used for all four analyzed miRNAs, unadjusted and adjusted for age and gender. The threshold for statistical significance was set to 5% (*p*-values lower than 0.05).

## Figures and Tables

**Table 1:** Descriptive characteristics for parameters of peripheral artery disease (PAD) patients overall and grouped by follow-up cardiovascular events (CVEs).

Parameter	Overall (*n* = 47)	No CVEs (*n* = 35)	CVEs (*n* = 12)	*p*-Value
Age (years)	63.5 ± 1.42	63.7 ± 1.74	62.9 ± 2.42	0.797
Gender (men, %)	43 (91.5)	31 (88.6)	12 (100)	0.221
Hypertension, *n* (%)	35 (74.5)	27 (77.1)	8 (66.7)	0.765
Hyperglycemia, *n* (%)	17 (36.2)	11 (31.4)	6 (50)	0.187
Dyslipidemia, *n* (%)	38 (80.8)	30 (85.7)	8 (66.7)	0.322
Coronary artery disease, *n* (%)	22 (46.8)	17 (48.6)	5 (41.6)	0.857
BMI (kg/m^2^)	26.1 ± 0.92	26.9 ± 1.01	22.9 ± 1.38	0.052
TC (mg/dL)	175.7 ± 6.54	176.8 ± 7.75	172.1 ± 12.45	0.756
TG (mg/dL)	129.7 ± 8.98	125.0 ± 9.48	149.2 ± 24.53	0.420
Glucose (mg/dL)	116.2 ± 4.56	116.0 ± 5.41	117.0 ± 8.48	0.604
LDL-C (mg/dL)	106.6 ± 5.63	109.9 ± 6.60	92.9 ± 8.50	0.236
HDL-C (mg/dL)	42.7 ± 1.54	43.6 ± 1.83	39.2 ± 2.4	0.297
HDL-C/LDL-C ratio	0.447 ± 0.028	0.446 ± 0.032	0.453 ± 0.062	0.559
APOA-I (mg/dL)	109.94 ± 9.30	117.8 ± 10.38	72.7 ± 15.0	0.028 *
APOE (mg/dL)	2.02 ± 0.17	2.00 ± 0.19	2.11 ± 0.46	0.836
APOA-I/APOE ratio	65.2 ± 5.96	70.3 ± 6.72	42.5 ± 8.81	0.025 *
PON1 (µg/mL)	1.68 ± 0.15	1.71 ± 0.17	1.26 ± 0.34	0.433
PON1 activity paraoxon (U/L)	263.5 ± 38.16	315.5 ± 43.46	143.2 ± 61.84	0.046 *
MPO (ng/mL)	48.14 ± 6.49	45.7 ± 7.87	57.6 ± 9.69	0.645
MPO activity (fmoles/min/mL)	226.0 ± 38.70	226.0 ± 49.10	255.0 ± 39.97	0.326
CP (µg/mL)	693.7 ± 43.15	626.7 ± 29.95	931.8 ± 144.78	0.002 *
CRP (mg/dL)	5.86 ± 1.60	4.27 ± 1.42	10.11 ± 4.28	0.022 *
LDH (U/L)	168.1 ± 14.41	164.9 ± 13.31	179.1 ± 47.37	0.539
AST (U/L)	24.91 ± 1.94	24.27 ± 1.24	26.67 ± 6.63	0.274
ALT (U/L)	21.24 ± 1.30	21.18 ± 1.36	22.08 ± 3.24	0.990
Age (years)	63.5 ± 1.42	63.7 ± 1.74	62.9 ± 2.42	0.797

BMI, body mass index; TC, total cholesterol; TG, triglyceride; LDL-C, low-density lipoprotein cholesterol; HDL-C, high-density lipoprotein cholesterol; APOA-I, apolipoprotein A-I; APOE, apolipoprotein E; PON1, paraoxonase 1; MPO, myeloperoxidase; CP, ceruloplasmin; CRP, C-reactive protein; LDH, lactate dehydrogenase; AST, aspartate transaminase; ALT, alanine transaminase; SEM, standard error of the mean. Data are given as means ± SEM, except for PON1, PON1 activity paraoxon, MPO, and MPO activity, which are given as medians ± SEM. Variations between the parameters of “No CVEs” vs. “CVEs” were analyzed by Mann–Whitney U-test or independent Student *t*-test (only for normally distributed variables) and considered statistically significant when the *p-*value was below 0.05 (marked with *).

**Table 2:** Associations among plasma miRNA levels and blood lipids, oxidative and inflammatory stress, and clinical parameters in multiple linear regression (MLR) analysis.

MLR	miR-142-3p		miR-223-3p		miR-155-5p		miR-92a-3p	
	β	*p*	β	*p*	β	*p*	β	*p*
**Model 1—Clinical data**								
Age	−0.054	0.891	−0.921	0.325	0.203	0.700	0.135	0.846
Gender	0.323	0.117	0.429	0.375	0.132	0.629	0.370	0.306
BMI	0.908	0.025	0.423	0.651	0.393	0.456	0.760	0.277
Obesity	−0.023	0.745	−0.200	0.235	−0.090	0.347	−0.121	0.335
Hypertension	0.092	0.484	0.365	0.244	−0.023	0.895	0.366	0.119
Dyslipidemia	−0.329	0.045	−0.079	0.837	0.210	0.332	−0.949	0.002
Hyperglycemia	−0.038	0.632	0.274	0.151	−0.014	0.897	−0.079	0.578
*R*^2^ (*p-*value)	0.845 (1.0 × 10^−13^)		0.132 (0.414)		0.723 (1.9 × 10^−11^)		0.517 (5.6 × 10^−6^)	
**Model 2—Blood lipids**								
TC	0.584	0.002	−0.286	0.482	0.252	0.271	0.407	0.252
TG	0.045	0.770	0.012	0.972	0.083	0.675	0.100	0.745
HDL-C/LDL-C	0.376	0.012	1.007	0.004	0.588	0.003	0.278	0.343
APOA-I/APOE	−0.057	0.679	−0.476	0.135	−0.030	0.866	−0.147	0.594
*R*^2^ (*p-*value)	0.850 (1.0 × 10^−13^)		0.206 (0.018)		0.751 (1.1 × 10^−13^)		0.396 (2.9 × 10^−5^)	
**Model 3—Oxidative and inflammatory parameters**								
PON1	0.300	0.044	0.121	0.739	0.325	0.132	0.088	0.760
PON1 activity	−0.171	0.179	−0.116	0.711	−0.024	0.896	−0.097	0.697
MPO	0.130	0.266	0.056	0.847	−0.034	0.842	0.572	0.015
MPO activity	0.052	0.675	−0.100	0.744	0.094	0.600	0.030	0.903
CP	0.645	9.4 × 10^−6^	0.123	0.706	0.340	0.081	0.166	0.523
CRP	0.095	0.349	0.182	0.470	0.276	0.066	0.017	0.933
*R*^2^ (*p-*value)	0.714 (1.9 × 10^−17^)		0.159 (0.764)		0.671 (1.02 × 10^−9^)		0.401 (5.4 × 10^−4^)	
**Model 4—Other parameters**								
LDH	0.556	2.3 × 10^−5^	−0.202	0.487	0.453	0.009	0.316	0.217
AST	0.077	0.568	−0.286	0.378	0.100	0.595	−0.132	0.641
ALT	0.324	0.018	0.766	0.020	0.323	0.088	0.412	0.148
*R*^2^ (*p-*value)	0.853 (1.0 × 10^−13^)		0.147 (0.039)		0.712 (1.4 × 10^−13^)		0.344 (6.2 × 10^−5^)	

BMI, body mass index; TC, total cholesterol; TG, triglyceride; HDL-C, high-density lipoprotein cholesterol; LDL-C, low-density lipoprotein cholesterol; APOA-I, apolipoprotein A-I; APOE, apolipoprotein E; PON1, paraoxonase 1; MPO, myeloperoxidase; CP, ceruloplasmin; CRP, C-reactive protein; LDH, lactate dehydrogenase; AST, aspartate aminotransferase; ALT, alanine aminotransferase. In the multiple linear regression models, plasma miRNA level was considered as a dependent variable, and the parameters included in the regression models were all considered as independent variables; “β” means standardized regression coefficients, “*p*” stands for statistical significance.

**Table 3:** Follow-up cardiovascular event (CVE) prediction by binary logistic regression using plasma miRNA levels. OR, odds ratio; CI, confidence interval.

Parameter	Wald χ^2^ Value	*p*-Value	OR (95% CI)	Model χ^2^ Value	Model *p*-Value
**Model 1**					
Plasma miR-142	4.58	0.032	1.1 × 10^6^ (3.25 − 3.78 × 10^1^)	24.75	5.65 × 10^−5^
Plasma miR-223	1.87	0.171	3.7 × 10^−2^ (3.26 × 10^−4^ − 4.17)		
Plasma miR-155	0.86	0.354	8.2 × 10^−2^ (4.20 × 10^−4^ − 16.17)		
Plasma miR-92a	5.29	0.021	4.0 × 10^−3^ (3.70 × 10^−5^ − 0.44)		
**Model 2**					
Plasma miR-142	3.19	0.034	6.9 × 10^6^ (0.221 − 2.20 × 10^14^)	25.47	2.80 × 10^−4^
Plasma miR-223	1.57	0.210	9.7 × 10^−3^ (6.95 × 10^−6^ − 13.54)		
Plasma miR-155	1.15	0.282	3.9 × 10^−2^ (1.05 × 10^−4^ − 14.47)		
Plasma miR-92a	3.97	0.046	2.2 × 10^−3^ (5.41 × 10^−6^ − 0.90)		
Age	0.22	0.635	1.05 (0.847 − 1.31)		
Gender	0.15	0.699	5.34 (1.10 × 10^−3^ − 2.60 × 10^4^)		

**Table 4:** Receiver operator characteristic (ROC) analysis for predictive potentials of individual plasma miRNA values and a combination of the four plasma miRNA values from peripheral artery disease (PAD) patients for follow-up cardiovascular events (CVEs). SE, standard error.

Area Under the Curve					
Test Result Variable(s) *	Area	SE ^a^	Asymptotic Sig. ^b^	Asymptotic 95% Confidence Interval	
				Lower Bound	Upper Bound
Plasma miR-142	0.861	0.068	0.007	0.727	0.995
Plasma miR-223	0.451	0.124	0.717	0.207	0.695
Plasma miR-155	0.632	0.152	0.325	0.335	0.929
Plasma miR-92a	0.396	0.099	0.437	0.202	0.589
All plasma miRNAs	0.924	0.050	0.002	0.825	1.000

* Adjusted for age and gender. ^a^ Under the nonparametric assumption. ^b^ Null hypothesis: true area = 0.5.

## Supplementary Materials

Supplementary materials can be found at https://www.mdpi.com/1422-0067/21/24/9600/s1.

## Abbreviations

ALT	alanine transaminase
APOA-I	apolipoprotein A-I
APOE	apolipoprotein E
AST	aspartate transaminase
BLR	binary logistic regression
BMI	body mass index
CAD	coronary artery disease
CP	ceruloplasmin
CRP	C-reactive protein
CVEs	cardiovascular events
DAVID	Database for Annotation, Visualization, and Integrated Discovery
EC	endothelial cell
GO	Gene Ontology
HDL	high-density lipoprotein
LDL	low-density lipoprotein
LDH	lactate dehydrogenase
miRNA	microRNA
MLR	multiple linear regression
MPO	myeloperoxidase
mRNA	messenger RNA
PAD	peripheral artery disease
PON1	paraoxonase 1
ROC	receiver operator characteristic
SEM	standard error of the mean
TC	total cholesterol
TG	triglyceride
