# Supplementary material for: Increased miR-142 Levels in Plasma and Atherosclerotic Plaques from Peripheral Artery Disease Patients with Post-Surgery Cardiovascular Events
Source: Int J Mol Sci. 2020 Dec 16;21(24):9600. doi: 10.3390/ijms21249600 (PMC7766790; doi:10.3390/ijms21249600)
Supplement: Supplementary file 1 [file ijms-21-09600-s001.pdf]

Supplementary material

Increased miR-142 levels in plasma and atherosclerotic plaques from peripheral artery disease patients with post-surgery cardiovascular events

Teodora Barbalata [1,*], Oriana E. Moraru [2,*], Camelia S. Stancu [1], Yvan Devaux [3], Maya Simionescu [1], Anca V. Sima [1], Loredan S. Niculescu [1,#], on behalf of EU-CardioRNA COST Action CA17129

[1] Lipidomics Department, Institute of Cellular Biology and Pathology "Nicolae Simionescu" of the Romanian Academy, 8, B.P. Hasdeu Street, Bucharest 050568, Romania; teodora.barbalata@icbp.ro (T.B.); camelia.stancu@icbp.ro (C.S.S.); maya.simionescu@icbp.ro (M.S.); anca.sima@icbp.ro (A.V.S.)

[2] Emergency Clinical Hospital "Prof. Dr. Agrippa Ionescu", 149 I.C. Brătianu Street, 077015 Baloteşti, Ilfov County, Romania; orianaelenamoraru@yahoo.com (O.E.M.)

[3] Cardiovascular Research Unit, Luxembourg Institute of Health, L-1445 Strassen, Luxembourg; yvan.devaux@lih.lu (Y.D.)

[*] These authors contributed equally to this work.

[#] Correspondence: loredan.niculescu@icbp.ro

Table S1. Bivariate Spearman nonparametric correlations between plaque miRNAs, plaque pri-miRNAs and plaque *Dicer* and *Drosha* mRNA expressions.

**Parameter**	**Spearman correlation**	**Plaque miR-223**	**Plaque miR-92a**	**Plaque miR-142**	**Plaque miR-155**
Plaque pri-miR-142	*R*	0.452	-0.278	0.837**	0.705**
	p value	0.091	0.297	3.53 x10^{-4}	0.007
Plaque pri-miR-223	*R*	0.493	-0.374	0.814**	0.448
	p value	0.062	0.154	7.02 x10^{-4}	0.108
Plaque pri-miR-155	*R*	0.366	-0.375	0.843**	0.566**
	p value	0.179	0.152	2.96 x10^{-4}	0.035
Plaque pri-miR-92a	*R*	0.125	-0.223	0.396	0.236
	p value	0.644	0.389	0.161	0.416
Plaque Drosha mRNA	*R*	-0.186	-0.003	0.391	0.227
	p value	0.508	0.991	0.187	0.434
Plaque DICER mRNA	*R*	0.023	-0.337	0.348	0.457
	p value	0.934	0.202	0.243	0.100
**Parameter**	**Spearman correlation**	**Plaque pri-miR-223**	**Plaque pri-miR-92a**	**Plaque pri-miR-142**	**Plaque pri-miR-155**
Plaque *Drosha* mRNA	*R*	0.777***	0.965***	0.444	0.912***
	p value	3.9 x10^{-4}	1.47 x10^{-9}	0.096	8.61 x10^{-7}
Plaque *Dicer* mRNA	*R*	0.274	0.095	0.054	0.108
	p value	0.305	0.725	0.846	0.688

*p <0.05, **p <0.01, *** p <0.001

Table S2. Bivariate Spearman's nonparametric correlations between plasma miRNAs levels and main clinical and biochemical parameters.

Parameter	Spearman correlation	Plasma miR-142	Plasma miR-223	Plasma miR-155	Plasma miR-92a
Age	*R*	-0.328*	-0.080	-0.139	-0.261
	p value	0.044	0.591	0.406	0.076
BMI	*R*	-0.263	0.075	-0.236	-0.153
	p value	0.291	0.740	0.345	0.485
TC	*R*	0.163	-0.146	-0.029	0.148
	p value	0.365	0.350	0.873	0.350
TG	*R*	0.186	-0.369*	-0.014	-0.064
	p value	0.299	0.015	0.937	0.683
Glucose	*R*	-0.216	-0.143	-0.177	-0.211
	p value	0.207	0.343	0.302	0.164
LDL-C	*R*	0.050	-0.100	-0.068	0.163
	p value	0.780	0.517	0.704	0.290
HDL-C	*R*	0.334	-0.138	0.208	0.097
	p value	0.053	0.379	0.239	0.539
APOA-I	*R*	-0.127	-0.152	0.063	-0.229
	p value	0.468	0.332	0.718	0.139
APOE	*R*	-0.077	0.016	-0.206	0.008
	p value	0.653	0.917	0.228	0.959
APOA-I/APOE ratio	*R*	0.008	-0.133	0.236	-0.134
	p value	0.963	0.395	0.172	0.392
PON1	*R*	0.111	-0.141	0.08	0.097
	p value	0.519	0.349	0.643	0.525
PON1 activity paraoxon	*R*	-0.174	-0.084	0.020	0.180
	p value	0.311	0.581	0.908	0.241
MPO	*R*	0.045	-0.206	-0.311	-0.136
	p value	0.796	0.174	0.065	0.372
MPO activity	*R*	0.063	-0.275	-0.123	-0.289
	p value	0.735	0.095	0.517	0.083
CP	*R*	0.038	-0.039	-0.194	-0.032
	p value	0.824	0.799	0.257	0.839
CRP	*R*	0.234	-0.181	-0.034	0.002
	p value	0.349	0.420	0.893	0.993
LDH	*R*	0.493**	0.318	-0.003	0.066
	p value	0.008	0.067	0.987	0.719

BMI, body mass index; TC, total cholesterol; TG, triglycerides; LDL-C, LDL-cholesterol; HDL-C, HDL-cholesterol; APOA-I, apolipoprotein A-I; APOE, apolipoprotein E; PON1, paraoxonase 1; MPO, myeloperoxidase; CP, ceruloplasmin; CRP, C-reactive protein; LDH, lactate dehydrogenase. *p<0.05, **p<0.01.

Table S3. Primer sequences used for gene expression analysis by real-time PCR in atherosclerotic plaques from femoral arteries of PAD patients.

Gene	Primer sequence 5'-3'	Gene ID	Amplicon size (bp)
DICER	Forward 5’-TTAACCTTTTGGTGTTTGATGAGTGT-3’<br>Reverse 5’-GGACATGATGGACAATTTTCACA-3’	NM_177438.2	94
DROSHA	Forward 5’-CATGTCACAGAATGTCGTTCCA-3’<br>Reverse 5’-GGGTGAAGCAGCCTCAGATTT-3’	NM_013235.4	115
18S	Forward 5’-CTACCACATCCAAGGAAGGCA-3’<br>Reverse 5’-TTTTTCGTCACTACCTCCCCG-3’	NR_003286.2	71
